# Autocatalytic sets in a partitioned biochemical network

**DOI:** 10.1186/1759-2208-5-2

**Published:** 2014-03-03

**Authors:** Joshua I Smith, Mike Steel, Wim Hordijk

**Affiliations:** Biomathematics Research Centre, Department of Mathematics and Statistics, University of Canterbury, Christchurch, New Zealand; SmartAnalytiX.com, Lausanne, Switzerland

**Keywords:** Origin of life, Peptide-RNA world, Autocatalysis

## Abstract

**Background:**

In previous work, RAF theory has been developed as a tool for making theoretical progress on the origin of life question, providing insight into the structure and occurrence of self-sustaining and collectively autocatalytic sets within catalytic polymer networks. We present here an extension in which there are two “independent” polymer sets, where catalysis occurs within and between the sets, but there are no reactions combining polymers from both sets. Such an extension reflects the interaction between nucleic acids and peptides observed in modern cells and proposed forms of early life.

**Results:**

We present theoretical work and simulations which suggest that the occurrence of autocatalytic sets is robust to the partitioned structure of the network. We also show that autocatalytic sets remain likely even when the molecules in the system are not polymers, and a low level of inhibition is present. Finally, we present a kinetic extension which assigns a rate to each reaction in the system, and show that identifying autocatalytic sets within such a system is an NP-complete problem.

**Conclusions:**

Recent experimental work has challenged the necessity of an RNA world by suggesting that peptide-nucleic acid interactions occurred early in chemical evolution. The present work indicates that such a peptide-RNA world could support the spontaneous development of autocatalytic sets and is thus a feasible alternative worthy of investigation.

## Background

Understanding the origin of life on Earth is an important and fascinating problem [1]. In order to shed light on the structure of early replicators and their mechanism of formation, various experimental approaches have been explored [2–5]. Due to the enormity of the task, experimental work alone seems unlikely to answer the question, and this has motivated several theoretical investigations [6–9]. While one goal of theoretical work is to accelerate experimental progress (either in top-down construction of a minimal cell [10], or the spontaneous formation of a self-replicating protocell from abiotic precursor molecules), links between theory and experiment have been scarce. Naturally, theoretical models are simplifications of real chemistry, and while such simplification enables progress, it may limit the conversation between theorists and experimentalists until the models more accurately reflect the complexity of real biochemical systems.

The combinatorial and stochastic aspects of theoretical work on the origin of life mean mathematics has an important role to play. The intuitive analogy between sets of reacting compounds and directed graphs was the motivation for Bollobas and Rasmussen’s work on directed cycles in random graphs [11]. In previous work [8, 12–15], RAF theory has been developed as an effective tool for making progress on theoretical questions about the origin of life, based on initial work by Kauffman [7, 16]. In particular, it appears the emergence of collectively autocatalytic and self-sustaining sets of chemical reactions (RAF sets, defined later) is necessary for the origin of life to occur. Previous work has investigated the structure of such sets and the probability of their formation, leading to theoretical and empirical (simulation-based) results.

The general ideas behind RAF theory are not unique, and there are several related formalisms [6, 9, 17]. However, in some cases, questions within the RAF framework have proven tractable while an equivalent question posed within an alternative formalism has not, perhaps because of the simplicity of the RAF model. On the other hand, it has been suggested that such simplicity limits our ability to draw conclusions about “real” biochemical systems. However, the recent demonstration of the ability of RAF theory to link theoretical and experimental results [4, 18], together with the ongoing development of fresh theoretical ideas [19], suggests that this framework continues to enable progress.

In this paper, we present a biologically relevant extension to the well-studied polymer model, formalising a network of molecules in which there are two “independent” types of polymer, which are able to catalyse each others’ (and their own) reactions, but cannot combine to form hybrid polymers. The motivation for this is the nature of the interaction between peptides and nucleic acids in the metabolic networks of modern cells. The importance of an extension addressing this mutually catalytic arrangement was highlighted in Kauffman’s 1986 paper, in note (vii) (p. 14): *“An independent then symbiotic coexistence of autocatalytic protein sets and template replicative polynucleotides would obviously be useful in prebiotic evolution.”* (While the present work does not address the templating ability of nucleic acids, this aspect has been studied previously [14, 20]). Moreover, this extension is highly relevant in the light of recent experimental results from Li et al. [5]. In their paper, the authors propose that interactions between polypeptides and polynucleotides occurred very early in chemical evolution, providing an alternative to the hypothesis that life began in an RNA World [21]. The authors state *“The striking reciprocity of proteins and RNA in biology is consistent with our proposal: proteins exclusively catalyze nucleic acid synthesis; RNA catalyzes protein synthesis; and genetic messages are interpreted by the small ribosomal subunit, a ribonucleoprotein.”* The reciprocity described here provides a clear motivation for theoretical investigation into the properties of these “symbiotic” polymer systems.

We present theoretical results showing that RAF sets are just as likely to emerge in such systems as in those previously studied [14], and it turns out that the result holds even for a more general system in which the molecules are not necessarily polymers, a small amount of inhibition is allowed, and the amount of catalysis varies freely across the reaction network. In previous work, catalysis has been assigned randomly with equal probability between each molecule and each reaction. The current work shows that RAF sets remain highly probable even under heterogenous catalysis, which is what we might expect to find in real biochemical networks.

As a step toward increased chemical realism, we introduce the concept of a kinetic chemical reaction system, in which every reaction has an associated rate, and all molecules are lost via diffusion into the environment at a constant rate. We can in principle then search for RAFs in the system (as in previous work [8]) with the additional requirement that every molecule in the RAF must be produced at least as fast as it is used up or diffuses away - we call such an RAF a *kinetically viable RAF* (kRAF).

### Definitions

We will use the notation of Hordijk and Steel [8]. Consider a triple , where

- *X*={*x*_1_,*x*_2_,… } is a (finite) set of *molecular species* or *molecule types*;
- *F*⊂*X* is a distinguished subset of molecular species known as the *food set*, the set of all species initially available in the environment;
is a (finite) set of chemically allowed *reactions*;
- Each reaction  is an ordered pair (*A*,*B*), where *A*⊆*X* is a multiset of *reactants* and *B*⊆*X* is a multiset of *products*. We can represent a reaction as *a*_1_+*a*_2_+⋯+*a*_*n*_→*b*_1_+*b*_2_+…*b*_*m*_. Note that the reactants *a*_*i*_ are not necessarily distinct, and neither are the products *b*_*i*_. Also note that reversible reactions can be modelled as two (formally) separate reactions .

The triple  is therefore a set of molecular species together with the reactions that occur between them, intuitively visualised as a directed graph. For brevity, we will often use the term “molecule” in place of “molecular species” or “molecule type”. We also define *ρ*(*r*) to be the set of all distinct reactants of the reaction *r*, and *π*(*r*) to be the set of all distinct products of *r*. Then for any subset  of ,  and . Another useful concept will be the *support* of a reaction *r*, supp(*r*):=*ρ*(*r*)∪*π*(*r*). Similarly,  for any subset  of . Informally, the support of a set of reactions is the set of all molecules consumed or produced by those reactions.

**Figure 1:**
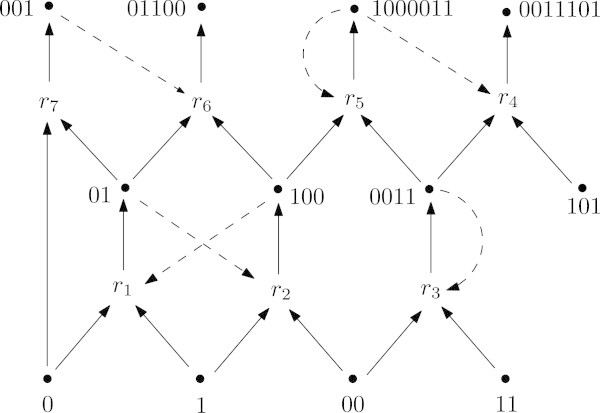
**A chemical reaction system.** A simple CRS within the binary polymer model, where the food set consists of all monomers and dimers. The subset {*r*_1_,*r*_2_,*r*_3_,*r*_5_} is the maximal RAF subset (the *maxRAF*), while {*r*_1_,*r*_2_}, {*r*_3_} and {*r*_1_,*r*_2_,*r*_3_} are smaller RAFs within the maximal RAF known as *subRAFs*. {*r*_1_,*r*_2_} is an example of an *irreducible* RAF, since no proper subset of it is an RAF. A reaction such as {*r*_3_}, which consists of a single autocatalytic reaction with food molecules as reactants, is sometimes called a ‘trivial RAF’.

We can equip the triple  with a *catalysation assignment*, where (*x*,*r*)∈*C* is understood to mean that the molecule *xcatalyses* reaction *r*: that is, *x* accelerates *r* but is unchanged by the reaction. A *chemical reaction system* (CRS) is now defined as a triple  together with a catalysation assignment *C*. We will denote a CRS by . Figure 1 shows an example of a CRS within the *binary polymer model*, defined by Kauffman [7] and well studied by Hordijk and Steel [8]. In this model, all molecule types are polymers over a 2-letter alphabet, and each reaction is either the ligation of two molecules into a longer polymer, or the cleavage of a single molecule into two shorter polymers.

The final important concept is that of the *closure* of the food set relative to a subset of reactions , denoted  and formally defined as the minimal subset *W*⊆*X* which contains *F* and satisfies *ρ*(*r*)∈*W*⇒*π*(*r*)∈*W* for all . Informally,  is the set of all molecules that can be built up from the food set using only reactions in  (ignoring catalysis).

Following [13], we say that a subset  of forms a *reflexively autocatalytic and food-generated set* (an RAF set) for provided that  is non-empty and that:

(i) All the reactants of each reaction in  are contained in  (food-generated);
(ii) For each , there exists (*x*,*r*)∈*C* such that  (reflexively autocatalytic).

We commonly use “*F*-generated” in place of “food-generated”, and “RAF” in place of “RAF set”. Informally, property (i) requires that the reactions in  must be able to sustain themselves from the food set alone. Property (ii) requires that every reaction in  must be catalysed, and furthermore that the catalysts must themselves be generated from the food set by that same set of reactions.

These definitions are intended to capture properties of chemical networks that may have been important in the emergence of early replicators. Uncatalysed reactions in general proceed extremely slowly. We require catalysis so that molecules accumulate in concentrations sufficient to perform useful biochemical tasks. Otherwise, they would diffuse away before being able to play any role in the emergence of the first replicator. Moreover, not only do catalysts greatly increase the reaction rates, they also lead to an equally dramatic reduction in the variance of the rate of reactions (*c.f.*[22], figure six); this last feature would seem to be important for obtaining some degree of synchronicity in both early and present-day metabolism. However, to allow the catalysts to come out of nowhere would be begging the question. So in addition, we require that the reactions generate their own catalysts from the food set (the set of all molecules available in a particular environment on early Earth).

The idea of a set being *F*-generated requires that no molecules are required as reactants before they have been produced. A set that fails to be *F*-generated could never have spontaneously built itself up from the molecules available on early earth (the food set), which is clearly a necessary condition for the development of early replicators from prebiotic chemistry. Note however that while the reflexively-autocatalytic requirement guarantees that an RAF set of reactions *eventually* produces a catalyst for every reaction, the definition of *F*-generated allows a reaction to proceed prior to the production of any of its catalysts. We consider this to be reasonable (and realistic) for the following reason. Reactions can proceed uncatalysed (albeit at a much lower rate), which may soon lead to the production of a catalyst for the reaction, establishing a positive feedback loop which quickly increases the rate of the reaction (consider the production of the molecule 0011 in Figure 1; this molecule is the sole catalyst for its own production). In previous work [13] we have studied a stronger type of autocatalytic set in which a catalyst must be present before a reactions can progress at all. These sets, referred to as *constructively autocatalytic* and *F*-generated sets (CAFs) have quite different properties to RAFs; indeed, they are less likely to appear spontaneously.

Figure 1 illustrates some ways in which a set can fail to be an RAF. The subset {*r*_1_,*r*_2_,*r*_3_,*r*_5_,*r*_7_} fails to be reflexively autocatalytic (and so fails to be an RAF) since *r*_7_ is uncatalysed. In the subset {*r*_1_,*r*_2_,*r*_3_,*r*_5_,*r*_6_} all reactions are catalysed, however the catalyst of *r*_6_ is outside  (the reactions do not collectively generate all of their own catalysts), so this subset also fails to be reflexively autocatalytic. The subset {*r*_1_,*r*_2_,*r*_3_,*r*_4_,*r*_5_} is reflexively autocatalytic (since every reaction is catalysed, and all the catalysts are in ), but it is not *F*-generated, since the reactant 101 of *r*_4_ is not in the closure set (it cannot be created from the food set by the reactions {*r*_1_,…,*r*_5_}). However, the subset {*r*_1_,*r*_2_,*r*_3_,*r*_5_} is an RAF. In fact, it is the largest RAF in the system, equal to the union of all RAFs in the system. Such an RAF is referred to as the maximal RAF subset or the *maxRAF*.

Given any catalytic reaction system , there is a fast (polynomial-time) algorithm which determines whether or not contains an RAF, and if so the algorithm constructs the maxRAF [8]. We use this algorithm in section “Simulations of partitioned chemical reactions systems” to study the emergence of RAFs within simulations of the partitioned polymer system, defined in the following section.

Note that the definitions of a CRS and of an RAF do not explicitly include consideration of reaction rates or concentrations. Therefore, the RAF formalism cannot address the more specific question of whether or not a population of molecules can remain stable enough to catalyze its own growth from the food set and growth over time to allow reproduction of the set, issues that are obviously of interest in an origin of life scenario. For example, an RAF might include an exceedingly rare reaction, the rate of which could never support the growth of the system, or a very fast reaction, which depletes an essential molecule. However, this purely algebraic approach has allowed the development of several important results that would not have been easy to deduce from a more detailed model. Nonetheless, once an RAF set is discovered, it can then be checked for dynamical stability: previous work [15, 18] has involved molecular flow simulations of RAF sets using the Gillespie algorithm [23]. Also, in section “Kinetic RAF framework” we consider an extension of the formal RAF framework which does take reaction rates into account.

### Partitioned polymer system

**Figure 2:**
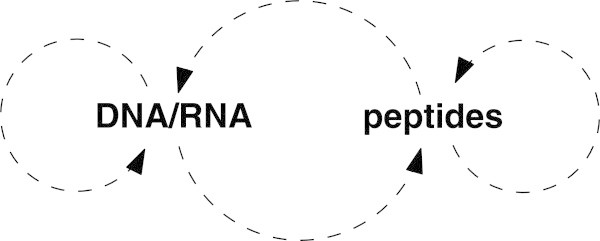
**Reciprocity of peptides and nucleic acids.** Schematic depicting the mutual catalytic dependence between nucleic acids and peptides in living systems, where a dashed arrow from X to Y indicates that there exist reactions involving molecules in Y which are catalysed by molecules in X. While all possible such arrows are present in the diagram, both groups of molecules are “closed” in the sense that there are no reactions combining nucleotide and amino acid monomers in the same polymer.

All modern life utilises at least two polymers for linking information to structure and function: nucleic acids (DNA/RNA) and peptides. Nucleic acids store and propagate genetic information, while peptides perform structural, catalytic and signalling roles *in vivo* in the form of proteins, enzymes and hormones. The interaction between peptides and nucleic acids is fundamental to the most important biochemical processes: peptides catalyse the replication of DNA and the synthesis of mRNA in transcription; at the ribosome, a combination of peptides and catalytic RNA molecules (ribozymes) catalyse the translation of mRNA, generating new peptide sequences. At the same time, each of these polymers catalyse reactions amongst themselves: for example, proteolytic enzymes catalyse the cleaveage of peptides, and a gene (DNA) could be considered to “catalyse” transcription of mRNA by acting as a template (Figure 2). Despite the mutual catalytic dependence of nucleic acids and peptides in living systems, these polymers are independent in the sense that there are no “hybrid” polymers containing both nucleotide and amino acid monomers^1^^2^. In order to formalise these properties we introduce the following generalisation of the well studied polymer model [8].

Consider a triple  within the polymer model. Let *X*, and *F* be partitioned as *X*={*X*_1_,*X*_2_},  and *F*={*F*_1_,*F*_2_}, where

- *X*_1_,*X*_2_ are disjoint sets of polymers;
- *F*_1_⊂*X*_1_ and *F*_2_⊂*X*_2_ are disjoint sets of food molecules;
is a set of ligation and cleavage reactions such that supp.

A *partitioned CRS* is now defined as a triple (partitioned as above) together with a catalysation assignment *C*. We will use the word *module* to refer to the set of molecules *X*_1_ together with the associated reactions , and similarly for *X*_2_ and . Hence, a partitioned CRS consists of two modules, and catalysis can occur both within (*intra-modular*) and between (*inter-modular*) the modules (the specific pattern of catalysis will depend on the nature of *C*). Note however that due to the condition , there can be no reactions involving molecules from both *X*_1_ and *X*_2_. We also allow *X*_1_ and *X*_2_ to be sets of polymers over different sized monomer alphabets. For example, let the size of these alphabets be *k*_1_ and *k*_2_: then to model the interaction between a set of peptides (*X*_1_) and a set of RNA polymers (*X*_2_), set *k*_1_=20, *k*_2_=4.

**Figure 3:**
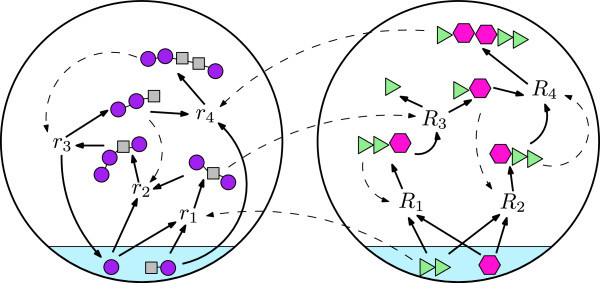
**A partitioned CRS.** A partitioned CRS within the binary polymer model. One set of molecules is built from the ‘square’ and ‘circle’ monomers, and the other is built from the ‘triangle’ and ‘hexagon’ monomers. The molecules at the bottom of the image comprise the food set and give rise to the other molecules via the ligation and cleavage reactions *r*_1_,…,*R*_4_. Dashed arrows indicate which molecules catalyse which reactions. In this case, the entire CRS is an RAF. Note that while there is intra- and inter-modular catalysis, there are no reactions involving molecules from both modules. This is emphasized by the enclosure of each module within a large circle: of course, in real systems, the molecules would be free to mingle.

Figure 3 shows a simple partitioned CRS within the binary polymer model. Previous work [8, 13] has demonstrated that in the standard, unpartitioned polymer model, RAFs are highly likely to be present in a CRS, given some mild requirements on the level of catalysis. Since the level of catalysis may vary across the network in the partitioned model, and since the partition makes the underlying structure of the reaction network qualitatively different, it is not obvious whether RAFs might be more or less likely to occur. This question is addressed more generally in the next section, where we prove a stronger result which is certainly sufficient to show that a partitioned CRS is just as likely to contain RAFs as an unpartitioned one. We will present the general result, before returning to the partitioned model.

## Results and discussion

### The probability of RAFs in general catalytic reaction systems

It was shown in [13] that for a CRS within the polymer model, the level of catalysis (expected number of reactions catalysed per molecule) necessary and sufficient to produce RAF sets with a given probability increases linearly with *n*, the maximum length of polymers in the system. Here we extend this result to a general CRS in which the molecules are not necessarily polymers, and we invoke slightly weaker assumptions by allowing the catalysation rates to vary between reactions; in a later section this approach also allows for a limited degree of inhibition.

For convenience, we will assume that the set of reactions is the disjoint union of two sets  and , where every reaction in  is of the form *a*+*b*→*c* (two reactants and one product), and  consists entirely of the corresponding reverse reactions *c*→*a*+*b*, so that . We refer to the reactions in  as ‘forward’ reactions. Thus pairs of corresponding reactions from  and  can be considered as a single reversible reaction. We will also assume that a molecule catalyses  if and only if that molecule also catalyses the corresponding , which reflects the reality of biological catalysis. These assumptions can be weakened, but doing so complicates slightly the statement and proofs of the results that follow, and they apply readily to the partitioned system that we study, as do the further conditions listed below.

In our generalised model we make two main assumptions concerning catalysation:

- (C1) The events  that molecule *x* catalyses (forward) reaction *r* are independent across all pairs .
- (C2) For some constant *K*≥1, the expected number of molecular species that catalyse any reaction is at most *K* times the expected number of molecular species that catalyse any other reaction.

Note that (C1) allows different molecule types to catalyse different numbers of reactions in expectation, since the probability that molecule type *x* catalyses reaction *r* can vary according to both *x* and *r* (in [13] it was assumed that the probability of  depends only on *x*, not on *r*).

Before stating the main result of this section, we require the following definition. We say that a triple  has a *species stratification* if and only if there is a nested sequence *α*_1_⊆*α*_2_⊆⋯⊆*α*_*m*_=*X* such that the following conditions hold: (i) *F*=*α*_*t*_ for some *t*<*m*; (ii) If the reaction *f*→*a*+*b* is in where *f*∈*F* then *a* and *b* are also elements of *α*_*t*_; (iii) The number of forward reactions involving any two food molecules as reactants is at most some fixed constant *M*; (iv) if we let *X*(1):=*α*_1_ and *X*(*s*):=*α*_*s*_−*α*_*s*−1_ for *s*∈{2,…,*m*} then:

- (S1) The number of molecules in *α*_*s*_ grows no faster than geometrically with *s*. That is, |*X*(*s*)|≤*k*^*s*^ for some fixed *k*≥1, for all *s*∈{1,…,*m*};
- (S2) Every molecule in *X*(*s*) can be constructed from molecules in *α*_*s*−1_ by a number of forward reactions that grows at least linearly with *s*−1. More precisely, for some fixed *ν*>0, the following holds: For each *s*∈{*t*+1,…,*m*}, and for all *x*∈*X*(*s*) we have:

We now show that for any triple  the probability that  (where the random assignment *C* satisfies (C1) and (C2)) has an RAF (denoted ) is, under certain conditions, determined by how the average catalysation rate compares to the simple ratio of the total number of forward reactions to the total number of molecules.

Let  be the average expected number of forward reactions that are catalysed by a molecular species (averaged over all molecular species in *X*). That is: 

The proof of part (a) of the following theorem is presented in the Appendix; part (b) follows immediately from a stronger result stated later (Theorem 2) and the proof of that later result is also in the Appendix

#### Theorem 1.

For any triple  that has a species stratification, consider the random CRS  formed by an assignment of catalysation (*C*) under any stochastic process satisfying (C1) and (C2).

- If  then the probability that there exists an RAF for is at most *ϕ*(*λ*), where  as *λ*→0, and where *τ* is a constant dependent only on *k* and *t*.
- If  then the probability that there exists an RAF for is at least 1−*ψ*(*λ*), where  exponentially fast as *λ*→*∞*.

The results in section “Simulations of partitioned chemical reactions systems” show that as the level of catalysis is increased past some threshold there is a transition in the probability of the existence of RAFs. This is to be expected as it is well known in combinatorics that every monotone increasing property of subsets of a set has an associated threshold function [24]. Consideration of the definitions of reflexively autocatalytic and *F*-generated reveals that the RAF property is monotone on the subsets of the set of possible catalysis arcs from molecules to reactions in a CRS, so the RAF property has a threshold function. In the original binary polymer model, the threshold function for catalysis is linear in *n* (the maximal sequence length). However, in the more general setting considered here, molecules do not come equipped with a intrinsic length. Nevertheless, Theorem 1 shows that the ratio of ‘reactions-to-molecules’ plays essentially the same role as *n* in a threshold function for the RAF property.

### Remarks

- The proof of part (b) involves the construction of an RAF involving every molecule in *X* (that is, ). However, in general, this RAF will involve only a subset of the reactions in .
- In general, the definition of a species stratification seems rather artificial: while a CRS within the simple (unpartitioned) polymer model naturally admits a species stratification (since we just let *α*_*s*_ be the set of all polymers up to length *s*), it would be a non-trivial exercise to find a species stratification for a CRS with molecules that are not polymers. Nevertheless, Theorem 1 shows that the molecules in a CRS being polymers is sufficient but not necessary, and we will see shortly that in the partitioned polymer model a species stratification also applies.

### The probability of RAFs in a partitioned CRS

In light of Theorem 1, in order to show that the same linear catalysis requirement that applies for an unpartitioned CRS holds for a partitioned one, we need only show that a partitioned CRS has a species stratification, and construct a set *C* satisfying (C1), (C2). In what follows, we will consider a partitioned CRS that satisfies the same assumptions that were made in the proof of Theorem 1 (i.e. , and corresponding reactions from  and  are always catalysed together). Also, let a molecule  belong to  if and only if the corresponding  does too, and let  denote the subset of all forward reactions in . Applying similar restrictions to , we thus consider a partitioned CRS in which is the disjoint union of four sets;  and , so that each module consists of an equal number of forward and reverse reactions together with the associated molecules (of course, the modules may contain different numbers of reactions to each other).

In previous work [19], *C* was often generated by randomly assigning catalysis as follows: let each element of  (and the corresponding element of ) be included in *C* with some fixed probability *p*. When studying metabolic network data from real organisms, we might expect to find that this uniform model does not match the observed pattern of catalysis: for example, it might be the case that peptides tend to catalyse more reactions involving other peptides than reactions involving nucleic acids. To allow for this possibility in a partitioned CRS, we allow the likelihood of catalysis to vary depending on both the nature of the catalyst and the nature of the molecules involved in the reaction. Specifically, we define the matrix **P** where, for any molecule *x*∈*X*_*i*_ and any reaction , the probability that *x* catalyses *r* (and the corresponding reverse reaction) is given by the *ij*th entry of **P**. For example, in a CRS generated using the matrix 

 we would expect to observe around ten times more catalysis within modules than between them, and twice as much catalysis of reactions in  by molecules in *X*_2_ than of reactions in  by molecules in *X*_1_.

In what follows, consider a partitioned CRS  which is *complete*: that is, both *X*_1_ and *X*_2_ contain every possible polymer up to length *n*_1_ and *n*_2_ (respectively), and  (respectively ) contains every possible forward and reverse reaction between the molecules in *X*_1_ (respectively *X*_2_). Let *F*_1_ (respectively *F*_2_) be all the molecules in *X*_1_ (respectively *X*_2_) up to some length *t*< min{*n*_1_,*n*_2_}. Finally, for a molecule *x*∈*X*, let |*x*| denote the length of *x* (i.e. the number of monomer units in *x*).

For *X*_1_, define the stratification 

 where *α*_*s*_ consists of all the molecules in *X*_1_ such that 1≤|*x*|≤*s*. It will prove useful to define *X*_1_(1):=*α*_1_ and for *s*∈{2,…,*n*_1_}, *X*_1_(*s*):=*α*_*s*_−*α*_*s*−1_. Similarly for *X*_2_, define the stratification 

and let *X*_2_(*s*) be defined similarly to *X*_1_(*s*). Note that . Defining *n*_min_:= min{*n*_1_,*n*_2_} and *n*_max_:= max{*n*_1_,*n*_2_}, these stratifications are combined into a single stratification *γ*_1_,*γ*_2_…, of the set *X* as follows:

- for 1≤*s*≤*n*_min_, *γ*_*s*_:=*α*_*s*_∪*β*_*s*_;
- for *n*_min_<*s*≤*n*_max_,

Note that *F*=*γ*_*t*_, which is condition (i) in the definition of a species stratification; conditions (ii) and (iii) also clearly hold (with *M*=2 for condition (iii)), so it remains to establish condition (iv), namely that the stratification satisfies (S1) and (S2). Define *X*(1):=*γ*(1) and for *s*∈{2,…,*n*_max_},*X*(*s*):=*γ*(*s*)−*γ*(*s*−1), and consider the size of each set *X*(*s*). Since |*X*(*s*)| does not exceed  for any value of *s*, (*k*_1_+*k*_2_)^*s*^ is strictly greater than |*X*(*s*)| for all *s*∈{1,…,*n*_max_}, so the partitioned CRS satisfies (S1). To see that it also satisfies (S2), we need only note that for any molecule type *x*∈*X*(*s*) where *s*∈{*t*+1,…,*n*_max_}, |*x*|=*s*, so there are a maximum of *s*−1 ways *x* could be constructed from shorter molecule types (i.e. molecule types in *γ*_*s*−1_). Since  and  are both complete, every such reaction exists and there are in fact precisely *s*−1 forward reactions generating *x* from *γ*_*s*−1_, so take *ν*=1. We conclude that the complete partitioned CRS has a species stratification.

It remains to show that the catalysation assignment *C* described above satisfies (C1), (C2). For each pair , the probability that *x* catalyses *r* (and the corresponding reverse reaction) is dependent only on which module *x* and *r* belong to, so (C1) clearly holds. The following expression gives the expected number of species that catalyse any given reaction: 

 where *c*_*i*_ is the expected number of species in *X* that catalyse any given reaction in . Noting that *p*_*ij*_∈[ 0,1] and that 

clearly *c*_1_,*c*_2_ are finite. Hence taking *K* = max{*c*_1_/*c*_2_,*c*_2_/*c*_1_} shows that (C2) holds also. We conclude that Theorem 1 applies to a partitioned CRS.

### Simulations of partitioned chemical reactions systems

Previous simulations of chemical reaction systems [8, 14] have focussed on those which are *complete* (*X* contains every molecule up to some maximum length *n*, and contains every possible cleavage/ligation reaction between the molecules of *X*) and those in which catalysis is assigned randomly such that every molecule has the same fixed probability of catalysing any reaction. In [13, 14], it was shown both theoretically and computationally that in a ‘classic’ CRS with only one module, the level of catalysis (expected number of reactions catalysed per molecule) necessary and sufficient to generate RAFs with a given probability (e.g. 0.5) increases linearly with *n*. Furthermore, simulations show that the linear relationship is not steep: when *n*=10, the required level of catalysis is around 1.29, and when *n*=20, the required level of catalysis increases only to 1.48 [14]. Based on the finding that many enzymes catalyse multiple reactions [25], and the results of a recent search for RAF sets in the metabolic network of *E. coli* (Sousa FL, Hordijk W, Steel M, Martin W: Autocatalytic sets in the metabolic network of *E. coli*, in preparation)., this level of catalysis appears to be biologically feasible. Hence, the above results suggest that RAFs might be expected for real biochemical polymer networks, even under a random assignment of catalysis.

**Figure 4:**
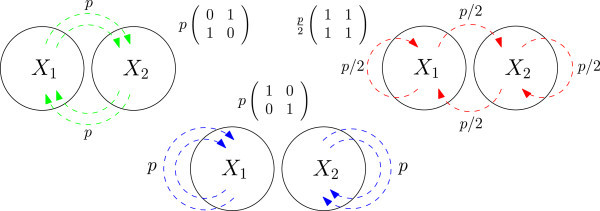
**Three models of catalysis in a partitioned CRS.** Visual representations of the three systems investigated via simulations: inter-modular (left), intra-modular (centre) and uniform (right). Labelled arrows from *X*_*i*_ to *X*_*j*_ indicate that molecules in *X*_*i*_ catalyse reactions involving molecules from *X*_*j*_, with some non-zero probability (given by the label). The catalysis matrix **P**, which depends on the parameter *p*, is shown for each system. It can readily be shown that the overall level of catalysis (expected number of reactions catalysed per molecule) is the same in all three scenarios.

Theorem 1 assures us that the linear increase in the required level of catalysis seen in the original model also applies to the partitioned model. However, it is not obvious whether or not the same realistic level of catalysis will be seen in the latter. In particular, because the partitioned model is highly flexible in terms of possible patterns of catalysis (forms of the matrix **P**), it is interesting to ask how the pattern of catalysis affects the probability of RAF formation. In order to address this question, we simulated a partitioned CRS in which each module is complete, with *k*_1_=*k*_2_=2, *n*=10, and the food set consists of all monomers and dimers. This CRS was simulated under three different catalytic assignments (Figure 4). In order to isolate the effect of the pattern of catalysis on the probability of RAF formation, the overall level of catalysis is constant across all three scenarios for any given value of *p*. We generated 500 instances of each model at a range of values of *p* (corresponding to a range of levels of catalysis) and searched for RAFs using the algorithm from [8]. Analyses were performed on an IBM Power755 cluster comprising 13 nodes, each with 32 CPUs running Linux 11.1 (a total of 416 CPUs).

**Figure 5:**
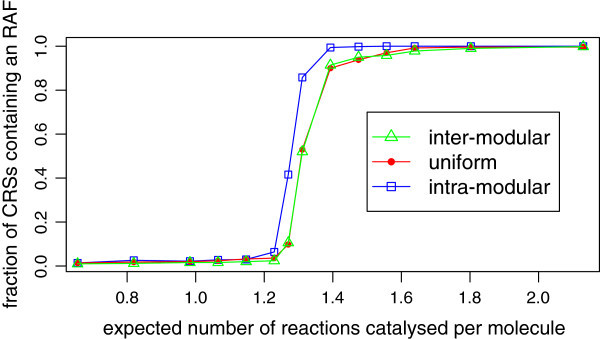
**Emergence of RAFs in a partitioned system with** ***n=10*** **.** Plot showing how the proportion of CRSs containing an RAF depends on the level of catalysis, for each of the three models. The maximum length of polymers (*n*) is 10 and the food set consists of all monomers and dimers. The fraction of CRSs containing an RAF is from 500 instances of the model.

Figure 5 shows, at each level of catalysis, the fraction of the 500 instances which were found to contain an RAF, for each of the three models investigated. All three models display a sharp transition in the probability of RAF formation as the level of catalysis increases, familiar from simulations of classic CRSs [8]. The uniform and inter-modular models display apparently identical results. The level of catalysis required to give 50% probability of RAF formation in these models (≈1.3) is slightly higher than that in the intra-modular model (≈1.25), indicating that during this transition, RAFs are slightly more likely in a CRS with only intra-modular catalysis than a CRS with some catalysis between modules. When the level of catalysis is 1.29, around 75% of instances of the intra-modular model contain an RAF, which is to be expected: the same level of catalysis in an unpartitioned CRS with *n*=10 gives 50% probability of RAF formation [14], and since here the intra-modular model essentially consists of two independent copies of the unpartitioned CRS, the probability of finding an RAF is 1−(1−0.5)^2^=0.75.Figure 5 also shows that, as the level of catalysis is increased past the transition level, the fraction of instances containing an RAF in the uniform and inter-modular models approaches 100% slower than in the intra-modular model. However, by the time the catalysis level has reached 1.7, all three models produce RAFs close to 100% of the time. These results indicate that the pattern of emergence of RAFs in partitioned chemical reaction systems is very similar to that in previously studied systems. Moreover, it is clear that even under widely varying patterns of catalysis, partitioned systems develop RAFs with high probability.

The uniqueness of the results from the intra-modular model suggests that the property unique to this model – the complete absence of inter-modular catalysis – has a discrete effect on the probability of RAF formation. Note that the uniform and inter-modular models both have inter-modular catalysis, but the latter has twice the level of the former, as well as a lack of intra-modular catalysis. Despite these difference, their results appear to be identical. Taken together, these results suggest that the presence or absence of inter-modular catalysis has more of an effect on the probability of RAF formation than the actual level of inter-modular catalysis.

**Figure 6:**
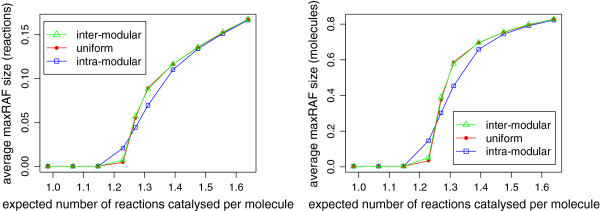
**Size of the maxRAF in a partitioned system with** ***n=10*** **.** Plots showing how the average number of reactions and number of molecules in the maxRAF change as the level of catalysis moves through the transition range, expressed as a proportion of the total number of reactions and molecules in the CRS. Averages are taken over 500 instances of each model where *n*=10 and the food set consists of all monomers and dimers. Instances containing no maxRAF were excluded from the calculation of the average, hence the data points that appear close to zero indicate a small but non-zero average size.

Despite the overall similarities in the pattern of emergence of RAFs between all three models, Figure 6 shows that the dependence of the size of the maxRAF on the level of catalysis is qualitatively different depending on the pattern of catalysis. At low catalysis levels, all three models tend to contain only RAFs consisting of a single reaction, catalysed by one of its own reactants or products. At the threshold level of catalysis at which all 3 models begin to develop RAFs with higher probability, the number of reactions contained in the maxRAF in the intra-modular model increases faster than in the other models (which again display very similar results). However, after a short delay, the number of reactions in the latter models rapidly increases, matching the equivalent value in the intra-modular model and then exceeding it. As the level of catalysis is increased further past the transition point, the rate of growth in the uniform and inter-modular models gradually decreases again, and all 3 models appear to converge on the same values of the average maxRAF size. This asymptotic behaviour makes sense: at higher levels of catalysis, the module to which any particular catalyst belongs has less bearing on whether or not the reaction in question is part of an RAF set, as the network becomes “saturated” with catalysis. Figure 6 also shows how the average number of molecules contained in the maxRAF (expressed as a proportion of all the molecules in *X*) depends on the level of catalysis (more formally this is , where  is the maxRAF). The pattern of growth is similar to that seen in the number of reactions. However, one important contrast is that, while the maxRAF quickly grows to involve the majority of the molecules in *X*, at a given level of catalysis it contains only a relatively small proportion of the reactions in . Thus as the level of catalysis is increased beyond that shown in Figure 6, we should expect the average proportion of molecules involved in the maxRAF to quickly approach 1.0, while the average proportion of reactions in the maxRAF continues to increase linearly. Not until a much higher level of catalysis will the maxRAF contain 100% of the reactions in the system. These results make sense, since the number of possible reactions in a polymer system is *O*(*n*2^*n*^), while the number of molecules is *O*(2^*n*^).

**Figure 7:**
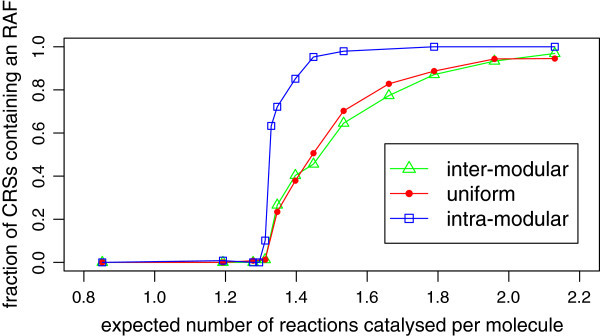
**Emergence of RAFs in a partitioned system with** ***n=15*** **.** Plot showing how the proportion of CRSs containing an RAF depends on the level of catalysis, when the maximum length of polymers in the system (*n*) is 15. The fraction of CRSs containing an RAF is from at least 120 instances of the model.

**Figure 8:**
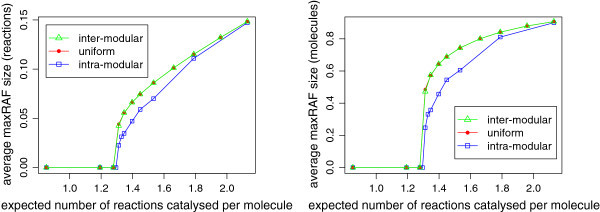
**Size of the maxRAF in a partitioned system with** ***n=15*** **.** Plots showing how the average number of reactions and average number of molecules in the maxRAF change as the level of catalysis is increased, expressed as a proportion of the total number of reactions and molecules in the CRS when *n*=15. Instances containing no maxRAF were excluded from the calculation of the average.

Overall, the above results show that when *n*=10, a partitioned CRS behaves very similarly to a classic CRS in terms of RAF emergence. In order to address the question of whether this is true for general values of *n*, we repeated the experiments at *n*=15 (Figures 7 and 8). For each of the three models, the level of catalysis required to attain a given probability of RAF formation is higher, which is to be expected given previous theoretical and experimental work on the original unpartitioned model. However, while the intra-modular model undergoes a sharp transition similar to the *n*=10 case, both the uniform and inter-modular models undergo a more gradual increase in the probability of RAF formation as the level of catalysis increases from around 1.3 up to 2.0 (Figure 7). Once again, the latter two models exhibit almost identical results, which is surprising given the difference in their pattern of catalysis. The level of catalysis required to give a 50% probability of RAF formation in the uniform and inter-modular models has increased from around 1.3 (*n*=10) to 1.45 (*n*=15), while the increase in the same figure for the intra-modular model is smaller, going from around 1.25 to around 1.32. However, the increased level of catalysis in the uniform and inter-modular models remains chemically realistic. Figure 7 also suggests that as the level of catalysis is further increased, the fraction of CRSs containing an RAF for these models will approach 1 monotonically, as observed for *n*=10 (Figure 5).

Figure 8 shows how the average size of the maxRAF (in terms of number of reactions, and number of molecules) changes as the level of catalysis is increased. Whereas for *n*=10 the maxRAF initially grew most quickly for the intra-modular model, these plots do not show the same early growth spurt: instead, all models appear to begin the transition at around the same level of catalysis. It is possible that the resolution was not high enough to detect the phenomenon: simulating smaller increments in *p* and a greater number of instances around this transition zone may reveal that it still occurs at *n*=15. Other than this, the plots are similar to those in Figure 6. RAF sets in the uniform and inter-modular models grow faster both in number of reactions and number of molecules, and not until a level of catalysis around 2.0 does the intra-modular model catch up. This is much later than in the *n*=10 case, which is particularly interesting given that at this level of catalysis the intra-modular model is developing RAFs with higher probability than the other models (Figure 7).

### Discussion

We chose here to investigate only the cases when *n*=10 and *n*=15, since computational constraints limit the feasibility of repeating the experiments for more and/or larger values of *n*. However, inferences can be made about other values of *n*, especially in the light of Theorem 1, which shows that a linear increase (with *n*) in the level of catalysis is necessary and sufficient to maintain RAFs with a given probability in a partitioned CRS. After producing similar results to the above for further values of *n*, it would be interesting to use least squares regression to explicitly express the linear dependence (on *n*) of the level of catalysis required to give 50% probability of RAF formation for various patterns of catalysis, and compare these with the linear formulae produced in [14] for the original model. Based of Figures 5 and 7, we expect to see a steeper relationship for the uniform and inter-modular models than for the intra-modular model.

While all three models begin to develop RAFs with high probability above the threshold level of catalysis, it is clear that the intra-modular model develops RAFs somewhat more reliably (with higher probability at lower catalysis levels) than the other models. Furthermore, this difference is more apparent at *n*=15 than *n*=10, and in the light of the result of Theorem 1, the difference looks likely to become more marked as *n* increases. On the other hand, as pointed out by philosopher Roger White [26], the probability of a mechanism proposed to play a role in the origin of life may not be a sound metric by which to judge the validity of that mechanism (Elliott Sober makes a related argument in response to Richard Dawkins in [27] pp.50-51). In terms of RAF theory, this means that the probability of RAF formation might not be the best way to decide which models have the most potential to shed light on the origin of life question.

However, the results show another difference between the models that is worth noting. Figures 6 and 8 both suggest that the size of RAF sets is significantly lower in the intra-modular model than in the uniform and inter-modular models (excluding the brief window immediately around the threshold level of catalysis in which RAFs in the intra-modular model grow faster at *n*=10). This larger size of RAF sets in the uniform and inter-modular models is interesting: since RAF sets can often be decomposed into constituent RAFs (subRAFs), larger RAFs are likely to contain more of these autocatalytic subsets. It was suggested in [28, 29] that this modular structure might be important for the potential evolvability of RAF sets. Specifically, the ability of large RAF sets to gain and lose smaller subRAFs might be a mechanism by which RAF sets can evolve and compete with each other, a process which might favour characteristic combinations of subRAFs, in a primitive form of selection. This transition from a purely self-replicating set of molecules to a complex autocatalytic set which replicates imperfectly while remaining robust to changes in the environment is essential, if RAF sets are to give rise to a replicator capable of gradual, open-ended Darwinian evolution.

We have investigated three different patterns of catalysis. Due to the inherent flexibility of the partitioned model, there are various other qualitatively different patterns that could be explored. In each of the above systems, the catalysis matrix **P** is symmetric. Even with this restriction in place, there is a continuum between exclusively inter-modular and exclusively intra-modular catalysis, and we examined only the middle point and the two extremes of that continuum here. We expect to observe a similar pattern of RAF emergence in other systems, where both intra- and inter-modular catalysis occur, but not in equal amounts. Based on Figures 5 and 7, if we were to begin with an exclusively intra-modular system and gradually increase the level of inter-module catalysis (the off-diagonal entries of **P**), while holding the overall level of catalysis constant, we should expect to see a shift in the pattern of RAF development, becoming more like the uniform and inter-modular models examined here. This change should be complete by the time the catalysis becomes uniform, so must occur somewhere between ‘intra-modular’ and ‘uniform’ . It would be interesting to determine at what point this transition occurs, and how sharp it is. A further extension would be to investigate systems in which **P** is not symmetric: for example, where one module dominates as a source of catalysts for the system . Given the main motivation behind this investigation, and the observation that peptides appear to be far more catalytically active than nucleic acids [25], this particular extension seems highly relevant.

Based on structural complementarity between polypeptide and RNA helices [30] and more recent experimental work demonstrating high catalytic proficiency of ancestrally related primitive forms of enzymes involved in translation [5, 31, 32], Carter and colleagues have suggested that the interactions between polypeptides and RNA may have played a key role in early chemical evolution in a “peptide-RNA world”. Our theoretical results show that a system with two different types of polymer with reciprocity of function similar to that of proteins and RNA, produces autocatalytic sets at similarly realistic levels of catalysis to a simpler system composed of a single type of polymer (such as an RNA-world or system of peptides). Therefore, the results presented here suggest that the alternative scenario proposed by Carter and colleagues is feasible.

### Extensions: closure, inhibition and reaction rates

The current definition of an RAF is limited because it ignores inhibition and reaction rates. The latter is problematic because those reactions generating required reactants which proceed too slowly, or those which use up required reactants and proceed too fast, may prevent an RAF set from persisting in a dynamic environment. While the lack of inhibition and kinetics may be seen as a severe restriction, it is useful because it allows us to compute RAFs in polynomial time. These RAFs could then be examined to test if they are viable given known inhibition or reaction rate data.

Alternatively, we could build this into the definition of a stronger type of RAF and ask if there is an efficient algorithm to find them. In this section we explore the latter approach. We consider RAFs that are viable under reaction rates and show that determining whether or not they exist in an arbitrary catalytic reaction system turns out to be NP-complete.

Consideration of these factors (inhibition and reaction rates) requires distinguishing between RAFs that are ‘closed’ and those that are not (this distinction is not important in the absence of inhibition and dynamics). Thus we first introduce and discuss this property, before considering the definition and properties of RAFs that allow inhibition or reaction rates.

### Closed RAFs

Given a CRS , a subset  of is a *closed RAF* if and only if the following conditions hold:

(i) is an RAF;
(ii) for every  for which there is a pair (*x*,*r*)∈*C* such that , .

Informally, a closed RAF captures the idea that “any reaction that can occur, will occur”. If all the reactants and at least one catalyst of a reaction  are generated by the reactions in , then it seems reasonable to expect that the reaction *r* will occur, and so we should expect that *r* is included in . If *r* is not included, then it is natural to consider adding it to , in order that the extended set  comes closer to containing all the reactions for which it generates all the necessary molecules. In order to formalise this notion, we introduce the idea of the *closure* of an RAF, defined as the smallest closed RAF which contains the RAF. Given an RAF , we can construct its closure  as follows: let , and let *K*_*i*+1_=*K*_*i*_∪*L*_*i*_, where *L*_*i*_ is the set of all  such that there exists a pair (*x*,*r*)∈*C* and . Then,  is the final set *K*_*n*_ in the sequence of nested sets , where *n* is the first value of *i* for which *K*_*i*_=*K*_*i*+1_.

Note that an RAF  is a closed RAF if and only if . Note also that while the union of two RAFs is also an RAF, the union of two closed RAFs is not necessarily a closed RAF (though it is an RAF).

One notable property of closed RAFs is that, unlike RAFs that are not closed, we can reconstruct the network of reactions given only a “list” of the molecules involved in the network, as follows.

#### Lemma 1

A closed RAF  is determined entirely by the subset of molecules  and the CRS .

#### Proof

Consider the set  reconstructed from  as follows:

1. Add to  every reaction  for which .
2. Remove from  any reaction *r* for which there does not exist an  such that (*x*,*r*)∈*C*.

We will show that  by establishing the set inclusions  and . First, consider some . Clearly . It remains to show that there exists some  such that (*x*,*r*)∈*C*. Since  is an RAF for , by Lemma 4.3 of [8], , which together with the definition of *F*-generated and of the support implies that 1

By definition of reflexively autocatalytic, it follows from (1) that for all , there exists  such that (*x*,*r*)∈*C*. Therefore every reaction in  fits the criteria for inclusion in , and we conclude that .

Next consider some . Then by the rules of construction of ,  and there exists an  such that (*x*,*r*)∈*C*. By (1), such an *x* is in , and also by (1),  so certainly . Then since  is a closed RAF,  by definition. We conclude that , which together with the previous result proves that .

#### Corollary 1

If  is an RAF, then given only  and the CRS , we can construct its closure .

#### Proof.

If  is a closed RAF, then  so the assertion holds trivially by the previous lemma.

Hence suppose  is not closed. Then there is at least one reaction  such that there exists a pair (*x*,*r*^∗^)∈*C* and . Construct the set of reactions  from  (as in Lemma 1). Since we did not use the fact that the RAF was closed in the first part of the proof of the lemma, we can apply the same argument to see that .

Now consider some . Then by the rules of construction of , , and there exists some  such that (*x*,*r*)∈*C*. Then by Equation (1) in the proof of Lemma 1 (again, this applies since we did not assume the RAF was closed in that part of the proof),  contains every  such that there exists a pair (*x*,*r*)∈*C* and . At this point, we identify  with the set *K*_0_ and  with the set *K*_1_=*K*_0_∪*L*_0_ described in the preamble to Lemma 1. We can then follow the same process described in the preamble, constructing a sequence of nested sets , where *K*_*n*_ is by definition equal to .

### Inhibition

In order to discuss the impact of molecules inhibiting reactions, we begin with the following definitions.

Given a CRS  an *inhibition assignment* is a subset *I* of  where (*x*,*r*)∈*I* means that molecular species *xinhibits* reaction *r*. We say that a subset  of is an *I*-viable RAF for if and only if all of the following hold:

is an RAF for ;
is closed;
- No reaction in  is inhibited by any molecule in .

The motivation for insisting that  be closed is as follows: Suppose that  involves a reaction that is inhibited by some product *x*^′^ of a reaction *r*^′^ that is not in . Now if the reactants, and at least one catalyst of *r*^′^ are present as products of reactions in  (or elements of *F*) then there is no reason for *r*^′^ not to proceed and for *x*^′^ not to be produced. In that case , and any set containing it, would no longer be an RAF.

The concept of an RAF subject to inhibition was formalized and studied briefly in [13], but there condition (b) was not imposed. This paper established that the problem of determining whether or not a CRS contains an RAF that is *I*-viable for is an NP-complete problem. It is pertinent therefore to ask whether the addition of condition (b) alters this result, or affects the proof. In fact, it can be shown that it does not, since the reduction in [13] involves the construction of an RAF that is automatically closed.

It is also of interest to know how inhibition affects the probability of forming a viable RAF, when *I* is a random assignment. Notice that inhibition is a much stronger notion than catalysation - since if a reaction is inhibited by just one molecule, then no matter how many molecules might catalyse that reaction, it is prevented from taking place. Thus we might expect that even low rates of inhibition could be a major obstruction to the formation of a viable RAF. However, we show here that provided the inhibition rate is sufficiently small, Theorem 2 still holds. To state this we first formalize the model by extending (C1) and (C2) to the following three conditions (which reduce to (C1) and (C2) upon setting *ε*=0).

- (C1) The events  that *x* catalyses reaction *r*, and the events  that *x* inhibits reaction *r* are independent across all pairs (*x*,*r*) in .
- (C2) As stated previously near the start of section “The probability of RAFs in general catalytic reaction systems”.
- (C3) For some constant *ε*≥0, the expected number of molecular species that inhibit any given reaction is at most *ε*.

Notice that part (a) of Theorem 1 applies automatically to the more restrictive notion of an inhibition viable RAF. However part (b) does not, and here we present a stronger result, which implies Theorem 1(b) (upon taking *ε*=0). The proof of this theorem is presented in the Appendix.

#### Theorem 2.

Consider a CRS that satisfies the extended conditions (C1)–(C3), and has a species stratification. Suppose further that the inhibition rate *ε* in (C3) satisfies: , where  is the average (over all reactions) expected number of molecular species that catalyse each reaction.

- If  then the probability that there exists an RAF for is at least 1−*ψ*(*λ*), where  exponentially fast as *λ*→*∞*.
- When *ε*=0 (no inhibition) the factor of 2 in the numerator and denominator of *ψ*(*λ*) can be removed.

### Kinetic RAF framework

Here we extend previous work by introducing the concept of a kinetic CRS, in which every reaction has an associated rate, and all molecules diffuse away into the environment at constant rate. We then define a kinetic RAF, which, informally, is an RAF in which every molecule is produced at least as fast as it is lost (to diffusion, or by consumption in other reactions). This represents the idea that being able to build up a sufficient local concentration of molecules is a necessary condition for RAFs to form.

**Definition:** A *kinetic CRS* is a tuple  where , *F* and *C* are defined in the same way as for a simple CRS, and  is a *rate function*, where for each , *v*(*r*) is the *rate* of *r*.

For any subset , the *stoichiometric matrix* is the  matrix with rows indexed by the non-food molecule types involved in  and columns indexed by the reactions in , where  is the net number of molecule type *i* produced by reaction *j*. The *rate vector* lists the rates of each reaction in . Then,  is a vector of the net rates of production of each molecule type in supp. Let *δ*≥0 be the *diffusion rate*.

A subset  is a *kinetic RAF* (kRAF) if and only if the following properties hold (where **1** is a  column vector of 1s):

is an RAF for ;
is closed;
- The following inequality holds: 2

Note that we do not include food molecules in the rows of . An RAF  is not guaranteed to contain any reactions which generate food molecules, but will necessarily contain at least one reaction with at least one food reactant. In that case, if we were to include the rows corresponding to those food molecules, they would have only negative entries, causing the RAF  (which might otherwise satisfy the properties of a kRAF) to formally fail to be a kRAF.

The diffusion rate *δ* represents the rate at which molecules diffuse away into the environment. Diffusion is unavoidable in chemical systems, and as molecules diffuse away, their concentrations drop until they are no longer available to sustain local reactions. A CRS occurring in the ocean or a “pond” might have a larger *δ* than one occurring in a hydrothermal vent, which may in turn have a larger *δ* than a CRS confined within a lipid membrane [33].

The idea of searching for kRAFs within a kinetic CRS is related to the idea in chemical organisation theory (COT) of searching for self-sustaining chemical organisations within an algebraic chemistry [9]. The definitions of the stoichiometric matrix coincide, and the qualifying condition (2) for a kRAF is similar to the qualifying condition for an organisation to be self-sustaining [9] (however in COT there is no diffusion term; note that  is necessary but not sufficient for a subset  to be a kRAF). Furthermore, in COT the entries of the vector **v** are not fixed - we are free to choose a set of values that makes the system self-sustaining, and indeed the definition of self-sustaining is simply that such a set of values can be found. In contrast, the reactions rates in a kinetic CRS are pre-determined constraints within which we can (in principle) go looking for a subset  of reactions that satisfies (2). While we propose that this set up is more relevant to the origin of life, the following theorem shows that such a search is unlikely to be useful in general. We show that determining whether or not contains a kRAF is NP-complete when *δ*=0 (we expect a similar result applies when *δ*>0 but our proof, presented in the Appendix, applies to the zero- diffusion case).

#### Theorem 3.

Given a kinetic CRS  with diffusion rate *δ*=0, the problem of determining whether or not contains a kRAF is NP-complete.

The closely related problem in COT of deciding whether or not an algebraic chemistry contains an organisation is also NP-complete [34]. Although Theorem 3 shows that we cannot hope to efficiently find kRAFs within a kinetic CRS, it is easy to check (in polynomial time) whether or not a given RAF is a kRAF, and since RAFs can be found in polynomial time [8], it may be feasible to discover kRAFs in a kinetic CRS by first ignoring the rate function *v* and finding a sample of RAFs, then deciding whether or not any are viable under *v*.

One weakness of the kRAF concept is that reaction rates are fixed - in real systems, the rate of a reaction is a function of the concentrations of its reactants, catalysts and inhibitors. Although the concept of concentration currently has no direct meaning in the RAF framework, previous work has used dynamical simulations to study the changes in concentrations of molecules in small RAF sets [15, 18].

## Conclusions

Due to the utility of polymers in modern life, much of the theoretical and experimental work on the origin of life problem has focussed on system of polymers, and in [13] it was shown that the level of catalysis need only increase linearly as the number of molecules increases, in order to maintain a high probability of RAFs occurring. We have presented a generalisation of this result, showing that under mild assumptions, the same linear bound applies to a system in which the molecules are not necessarily polymers. Furthermore, partitioned systems were shown to support the development of RAFs similarly to typical systems containing only one type of polymer, and the effect of the pattern of catalysis on the emergence of RAF sets was explored. Previous research into template-based catalysis [14, 20] and recent work incorporating more realistic patterns of catalysis [35] have indicated that the emergence of RAFs is quite robust to the the structure of the underlying reaction system, a conclusion which this paper supports.

This research was performed in an effort to better understand the “symbiotic coexistence” of peptides and nucleic acids in living organisms, as well as the potential role of this reciprocity in early chemical evolution (as highlighted recently by [5]). While the results presented here are a far cry from deep insights revealing fundamental truths about the origin of life, this extension of previous work on chemical reaction systems represents an incremental gain in understanding, which can hopefully contribute to an eventual bigger picture. In particular, this paper supports the experimental work of Li et al. [5] and encourages further experimental work on the topic.

We have also introduced and studied two new concepts in RAF theory: closed RAF sets, and kinetic chemical reaction systems. A closed RAF set is an RAF set in the standard sense, with the additional property that “every reaction that can occur, does occur”. More specifically, this means that if the existing subset of reactions is able to produce all the reactants and at least one catalyst of a reaction outside of the subset, then that reaction should be included the subset. A closed RAF is a subset of reactions that has “absorbed” every such reaction.

The kinetic RAF framework was developed in response to criticism levelled at RAF theory for not accounting for the fact that reactions progress at different rates. Kinetics is a fundamental part of real chemistry, so while the strength of RAF theory perhaps lies in its simplicity, the development of a kinetic extension is appropriate. A centerpiece of previous RAF theory investigations has been the search algorithm from [8], which runs in polynomial time and which has allowed chemical reaction systems of various sizes and properties to be investigated computationally [8, 14]. Therefore, a similar algorithm for detecting kinetically viable RAFs inside a kinetic CRS would be a promising start for the development of a theory of kinetic RAFs. Unfortunately, a reduction from the NP-complete problem 3-SAT showed that detecting a kinetic RAF within a kinetic CRS is unlikely to be productive in general. However, it is possible to construct RAFs efficiently, and for each RAF found one can readily test whether it is also a kRAF and therefore potentially capable of true autocatalytic growth.

## Endnotes

^1^ Peptide nucleic acid (PNA) does exist, however this polymer has a backbone of N-(2-aminoethyl)glycine (AEG) monomers linked by peptide bonds, with nucleobases attached to each monomer, rather than being composed of both nucleotide and amino acid monomers. Interestingly, the recent discovery of AEG production in diverse taxa of cyanobacteria may suggest an information-carrying role for PNA in early life [36].

^2^ tRNA aminoacylation or “charging” involves the esterification of an amino acid monomer to the relevant tRNA, prior to translation at the ribosome. This is of course an example of a reaction which combines molecules from both “independent” sets.

## Appendix

### Proof of Theorem 1 and Theorem 2

Let *c*(*r*) denote the expected number of molecular species that catalyse reaction *r*, let  and  denote the lower and upper bounds on these values, respectively, and let  denote the average value. Then from the definition of  we have: 

 and (C2) furnishes the three inequalities: 3

Next we establish the following variation on a lemma from [13].

#### Lemma 2.

Consider a random CRS , satisfying (C1)–(C3). For a reaction  let *q*_*r*_ be the probability that either no species in *X* catalyses *r* or at least one species in *X* inhibits reaction *r*.

(i) *q*_*r*_≥1−*c*_*u*_,
(ii) *q*_*r*_≤ exp(−*c*_*l*_)+*ε*.

Let  denote the probability that species *x* catalyses reaction *r*, and let  denote the probability that *x* inhibits *r*. Note that 1−*q*_*r*_ is the probability that at least one species in *X* catalyses *r* and no species in *X* inhibits *r* and so, by condition (C1), we have: 4

Thus, 

 and  is the expected number of species that catalyse *r*, which by (C2) is at most *c*_*u*_. Thus, *q*_*r*_≥1−*c*_*u*_ which establishes part (i).

For part (ii) we have from (4): 

 and since 

 and 

(by (C2) and (C3)) we obtain the claimed inequality in part (ii).

To establish Theorem 1 part (a), observe that any RAF must contain at least one catalysed reaction that has its reactant(s) in *F*; we call such a reaction *primary*. By species stratification conditions (i), (ii) and (S1), the number of reverse reactions *f*→*a*+*b*, such that *f*∈*F* is bounded by a function of *k* and *t*; while by the species stratification conditions (i), (iii) and (S1), the number of forward reactions *f*+*f*^′^→*g* such that *f*,*f*^′^∈*F* is also bounded by a function of *k* and *t* (involving the constant *M* mentioned in (iii)). Thus the total number of primary reactions is at most a constant *τ* dependent only on *k* and *t*. By Lemma 2 (i) and condition (C1), the probability  that none of the primary reactions are catalysed satisfies: 5

Now, the probability that at least one of the primary reactions is catalysed is , and this is clearly a necessary (but not sufficient) condition for there to be an RAF for . It follows from (3) and (5) that: 

Consequently, if  then , and so we arrive at Theorem 1(a), with *ϕ*(*λ*)=1−(1−*λ*/*K*)^*τ*^.

To establish Theorem 2 (which implies Theorem 1(b)), let *q*_−_:= exp(−*c*_*l*_)+*ε*. By the upper bound on *ε* stated in the theorem (and the inequality  from (3)) we have: 6

By Lemma 2(ii), for any *s*≥*t* (recalling that *F*=*α*_*t*_), the probability that a species *x*∈*X*(*s*+1) cannot be produced from reactants in *α*_*s*_ is at most (*q*_−_)^*cs*^ (since by (S2) we know that there exist at least *cs* reactions producing *x* from reactants in *α*_*s*_, so the only way for *x* to fail to be produced is if each such reaction has either no catalyst in *X* or an inhibitor in *X*).

Let *N*_*s*_ denotes the number of species in *X*(*s*+1) which cannot be produced by catalysed and uninhibited reactions from reactants in *α*_*s*_. Then the expected value of *N*_*s*_ satisfies the inequality , and, by (S1) the term on the right is bounded above by *k*^*s*+1^(*q*_−_)^*νs*^. In particular, since , the probability (let us call it *W*_*s*+1_) that there exists at least one species in *X*(*s*+1) which cannot be produced from reactants in *α*_*s*_ satisfies: 7

Let us say a species in *X* is *problematic* if each reaction producing that species is either not catalysed by any molecule in *X* or is inhibited by at least one molecule in *X*. Then the probability that there exists a problematic species in *X* is , which, from (7), satisfies: 

 where the second inequality applies (6). Thus the probability that there are no problematic species in *X* is . A lower bound on this quantity is given by: 

Noting that there being no problematic species in *X* is a sufficient condition for to have an RAF  (indeed one with supp), we see that  is a lower bound on . Hence taking 

and noting that *ψ*(*λ*)→0 as *λ*→*∞*, part (b) follows (observe that this RAF is closed, since it involves all molecules in *X*). Finally, note that when *ε*=0 the inequality in (6) can be improved to  which eliminates the factor of 2. This completes the proof.

### Proof of Theorem 3

Firstly, given a kinetic CRS  and a subset  of it can be checked in polynomial time whether  is a kRAF for , and so the question of whether or not contains a kRAF is in the complexity class NP. We will show that this question is NP-complete for the case *δ*=0 by exhibiting a polynomial-time reduction from the NP-complete problem 3-SAT. Suppose we have an instance of 3-SAT, which is an expression *P* written in conjunctive normal form involving a set *Y*={*y*_1_,…,*y*_*n*_} of literals, and with each clause consisting of a disjunction of at most three variables (a literal *y*_*j*_ or its negation ). Thus we can write *P* in the form 

For example,  would be an instance of 3-SAT for *Y*={*y*_1_,*y*_2_,*y*_3_,*y*_4_}.

Here *T*(*i*) and *F*(*i*) are subsets of {1,…,*n*} that describe which elements of *Y* are in *C*_*i*_ as a literal or a negated literal (respectively). Since each clause has at most three variables, |*T*(*i*)|+|*F*(*i*)|≤3. We say that *P* has a *satisfying assignment* if there is a function *S*:*Y*→{true,false} so that for each clause *C*_*i*_ in *P*, there exists *j*∈*T*(*i*) for which *f*(*y*_*j*_)=true or a *j*∈*F*(*i*) for which *f*(*y*_*j*_)=false. In the example above, setting *S*(*y*_1_)=true, *S*(*y*_2_)=*S*(*y*_4_)=false, and *S*(*y*_3_) to be either true or false provides a satisfying assignment for *P*.

Given *P* we will construct a catalytic reaction system , food set *F*, and rate function *v* so that  has a kRAF if and only if *P* has a satisfying assignment.

We take *F*={*f*_1_,…,*f*_*n*_}, and let . Set 

The reactions, associated rates, and catalysts are described as follows: 89101112131415

First suppose that  contains a kRAF ; we will show that *P* has a satisfying assignment. Since  is an RAF, it is non-empty. Therefore, the molecule *T* must be produced, since every reaction in is catalysed by either *T* or some molecule that is produced from *T*. This in turn requires that for each 1≤*i*≤*k*, *θ*_*i*_ is produced, and therefore, for each 1≤*i*≤*k* there exists *j*∈*T*(*i*) such that *y*_*j*_ is produced or *j*∈*F*(*i*) such that  is produced. Furthermore, for each value of 1≤*j*≤*n*, at most one of the molecules  is produced, since otherwise by the closure property the *j*th reaction described by (15) would be contained in , which would destroy both *y*_*j*_ and  faster than either is produced and violate the rate property of the kRAF . A satisfying assignment *S* for *P* is now provided by setting *S*(*y*_*j*_) to be true (respectively false) if *y*_*j*_ is produced by some reaction in  (respectively not produced by some reaction in ). Note that *S* is a satisfying assignment even in the case where neither of  is produced for some *j*∈{1,…,*n*}, since in that case *S*(*y*_*j*_) can be chosen arbitrarily.

Conversely suppose that *P* has a satisfying assignment *S*; we will show that  contains a kRAF. Let  consist of reaction (14) together with the following reactions:

- for each *j*∈{1,…,*n*} such that *S*(*y*_*j*_)=true, include the *j*th reaction from (8), the *j*th reaction from (10), and every reaction from (12) such that *j*∈*T*(*i*);
- for each *j*∈{1,…,*n*} such that *S*(*y*_*j*_)=false, include the *j*th reaction from (9), the *j*th reaction from (11), and every reaction from (13) such that *j*∈*T*(*i*).

To show that  is a kRAF, we must show that it is a closed RAF which satisfies the rate requirement (Equation 2). It is easy to see that 16

so  is *F*-generated. Moreover, every reaction is catalysed by exactly one molecule from the set , and since this union is a subset of ,  is also reflexively autocatalytic and is therefore an RAF set.

 is closed if there are no reactions  such that there exists (*x*,*r*)∈*C* with . By the construction of ,  contains the following reactions:

- *f*_*j*_→*y*_*j*_ for each *j* such that *S*(*y*_*j*_)=false (catalysed by *y*_*j*_*T*);
for each *j* such that true (catalysed by );

(the catalysts of these reactions are not contained in )

- *y*_*j*_+*T*→*y*_*j*_*T* for each *j* such that *S*(*y*_*j*_)=false;
for each *j* such that ;
- *y*_*j*_→*θ*_*i*_ for each pair (*i,j*) with *j*∈*T*(*i*) and *S*(*y*_*j*_)=false;
for each pair (*i,j*) with *j*∈*F*(*i*) and *S*(*y*_*j*_)=true;

(Other than *T*, the reactants of these reactions are not contained in )

.

For each value of *j*, exactly one of the two reactants of this last reaction is contained in . Hence,  is closed.

It remains to show that  satisfies the rate condition from section “Kinetic RAF framework”. Recall that the rows of the stoichiometric matrix are indexed by the molecules in the set 

the elements of which are given by (16).

The molecules {*y*_*j*_:*S*(*y*_*j*_)=true} are each produced at rate *k*+1 from *f*_*j*_, used up at rate *ε*>0 to produce *y*_*j*_*T*, and used up at rate 1 by each of the reactions {*y*_*j*_→*θ*_*i*_:*j*∈*F*(*i*)}. Since there are *k* clauses, there are at most *k* values of *i* for which *j*∈*T*(*i*). Hence the overall rate of production of each molecule *y*_*j*_ is at least *k*+1−(*k*+*ε*)=1−*ε*>0, which satisfies the rate condition. A similar argument can be made to show that the molecules  also satisfy the condition.

The molecule *T* is produced at rate 1 by the reaction *θ*_1_+⋯+*θ*_*k*_→*T*, and used up at rate 0<*ε*<1/*n* by each of the *n* reactions forming *y*_*j*_*T* or . Hence the overall rate of production of *T* is guaranteed to be positive.

Consider the molecules *θ*_1_,…,*θ*_*k*_. *θ*_*i*_ is produced at rate 1 by each reaction from (12) or (13) that is included in , of which there are at least one (since *P* has a satisfying assignment). *θ*_*i*_ is also used up at rate 1 by reaction (14), hence the overall rate of formation of *θ*_*i*_ is non-negative.

Finally, noting that the molecules *y*_*j*_*T* and  are all produced at rate *ε*>0 and are not used by any reaction, we see that every molecule in  is produced at least as fast as it is used up. This shows that  is a kRAF, and so completes the reduction.
